# Extracellular vesicles in chronic kidney disease: diagnostic and therapeutic roles

**DOI:** 10.3389/fphar.2024.1371874

**Published:** 2024-03-13

**Authors:** Yixuan Zheng, Hui Wang, Xueying Li, Jing Xie, Junming Fan, Sichong Ren

**Affiliations:** ^1^ Chengdu University of Traditional Chinese Medicine, Chengdu, China; ^2^ Department of Nephrology, Hospital of Chengdu University of Traditional Chinese Medicine, Chengdu, China; ^3^ Chongqing Beibei Hospital of Traditional Chinese Medicine, Chongqing Hospital of the First Affiliated Hospital of Guangzhou University of Chinese Medicine, Chengdu, China; ^4^ Chengdu Medical College, Chengdu, China; ^5^ Department of Nephrology, First Affiliated Hospital of Chengdu Medical College, Chengdu, China; ^6^ Clinical Research Center for Geriatrics of Sichuan Province, Chengdu, China

**Keywords:** extracellular vesicles, exosomes, chronic kidney disease, biomarker, treatment

## Abstract

Chronic kidney disease (CKD) is a progressive disorder characterized by structural and functional changes in the kidneys, providing a global health challenge with significant impacts on mortality rates. Extracellular vesicles (EVs), are vital in the physiological and pathological processes associated with CKD. They have been shown to modulate key pathways involved in renal injury, including inflammation, fibrosis, apoptosis, and oxidative stress. Currently, the application research of EVs in the diagnosis and treatment of CKD is highly prevalent. However, there is currently a lack of standardized guidelines for their application, and various methodologies have advantages and limitations. Consequently, we present an comprehensive summary elucidating the multifaceted involvement of EVs in both physiological and pathological aspects in CKD. Furthermore, we explore their potential as biomarkers and diverse therapeutic roles in CKD. This review provides an overview of the current state of research on application of EVs in the diagnosis and therapeutic management of CKD.

## 1 Introduction

Chronic kidney disease (CKD) is a progressive disorder that results from various causes, typically defined as a reduction in renal function, an estimated glomerular filtration rate (eGFR) of less than 60 mL/min per 1.73 m^2^, or markers of kidney damage (albuminuria, haematuria, or abnormalities detected through laboratory testing or imaging) are present for at least 3 months (Webster et al., 2017). CKD is caused by various factors such as diabetes, hypertension, glomerulonephritis, which lead to irreversible impairment of renal function and structure (Levey et al., 2015). Typically, CKD gradually develops into end-stage renal disease (ESRD), leading to progressive uremia, electrolyte abnormalities, anemia, mineral and bone metabolism abnormalities and acid-base imbalance. Without treatment, it will inevitably result in death (Zarantonello et al., 2021). Currently, the intervention for CKD primarily focuses on two aspects: slowing disease progression and reducing further renal damage. Notably, four scientifically validated strategies have been applied to impede the progression of kidney disease including regulating hypertension, using renin-angiotensin-aldosterone system inhibitors, managing diabetes and hyperglycemia, as well as correcting metabolic acidosis (Vassalotti et al., 2016). The global prevalence of CKD in 2017 was nearly 700 million. The global burden of CKD increased significantly by 29.3% from 1990 to 2017 (Collaboration, 2020). Although current diagnostic and treatment methods for CKD are feasible, it remains a significant threat to human health. Therefore, the exploration of more sensitive biomarkers and effective treatment approaches continues to be a major challenge in diagnosing and treating CKD.

EVs are double-layer phospholipid vesicles secreted by cells and commonly found in various body fluids or cell culture. Typically, they can be classified into three distinct groups based on their biological origins: exosomes, microvesicles (MVs), and apoptotic bodies (ABs). In recent years, EV research has received extensive attention (Elsharkasy et al., 2020). The constituents of EVs are composed of proteins (e.g., heat-shock proteins, tetraspa-nins, and Alix), lipids (e.g., ceramide and cholesterol), and nucleic acids (e.g., DNA, mRNAs, and microRNAs). With a large number of these contents, EVs shuttle between cells and tissues, transferring signals and mediating micro-environmental communication in certain diseases (Lin et al., 2019; Li et al., 2020a; Li et al., 2020b; Wang et al., 2020b). Thus, EVs can possess significant physiological and pathological functions, including immune regulation, wound healing facilitation, and modulation of tumor initiation and progression (van Balkom et al., 2011).

Currently, due to their abundant sources and stable biological activities, EVs possess remarkable diagnostic value as a minimally invasive liquid biopsy for monitoring disease progression (Colombo et al., 2014). An increasing number of EV-related diagnostic biomolecules have been identified as potential biomarkers for a wide range of diseases, including cardiovascular diseases (Hafiane and Daskalopoulou, 2018), cancer (Fitts et al., 2019), central nervous system disorders (Kanninen et al., 2016), and CKD (Zhang et al., 2016). Additionally, the therapeutic effect of EVs has emerged as prominent area of research. EVs from various sources have been shown to ameliorate the pathological status as well as postpone the progression of many diseases including CKD (Ståhl et al., 2019; Corrêa et al., 2021; Cheng and Hill, 2022). It has been reported that a wide range of cell types such as fibroblasts, epithelial cells, blood cells, adipocytes, neurons, stromal cells, tumor cells, chondrocytes and mesenchymal stem cells (MSCs), exhibited the capability to secrete EVs (Kalluri and LeBleu, 2020). Many studies have reported multiple therapeutic roles of EVs in CKD by regulating pathways involved in renal injury, including inflammation, fibrosis, apoptosis and oxidative stress. Additionally, they also facilitate renal regeneration by promoting angiogenesis and cell proliferation (Nagaishi et al., 2016; Liu et al., 2018a; Ebrahim et al., 2018). In this review, we aim to elucidate the role of EVs in the pathogenesis of CKD and primarily focus on their potential as biomarkers and therapeutic agents for CKD.

## 2 Biogenesis and isolation methods of EVs

### 2.1 Biogenesis of EVs

The biogenesis of EVs is a highly complex process with heterogeneity defined by factors such as size, cargo composition, functional impact on recipient cells, and cellular origin. Exosomes are the most extensively studied EVs in recent years. In 1984, exosomes were first found in the supernatant of sheep erythrocytes cultured *in vitro* (Pan and Johnstone, 1984). Researchers noticed that certain vesicles could transfer unnecessary proteins between cells. The process of exosome generation involves two steps: first, the cell plasma membrane double invaginates, wrapping around extracellular components and cell membrane proteins to form early sorting endosomes (ESEs). Secondly, these ESEs fuse together to form intracellular multivesicular bodies (MVBs), which contain numerous intraluminal vesicles (ILVs). ILVs are then secreted as exosomes through fusion with the plasma membrane and exocytosis (Raposo and Stoorvogel, 2013).

MVs are a type of EV with diameters ranging from 100 to 1,000 nm that originate by budding from the plasma membrane. Unlike the early intracellular steps of exosome biogenesis, MV biogenesis begins with the plasma membrane budding directly outward (Clancy et al., 2021). The biogenesis mechanism begins with the outward budding and pinching of the plasma membrane (PM), which releases newly produced MVs straight into the extracellular environment (Muralidharan-Chari et al., 2009). Meanwhile, SCRT-dependent mechanisms and small GTPases may both be involved in this process (Sedgwick et al., 2015; Mathieu et al., 2019; Fang et al., 2024).

ABs are generated during the process of cell apoptosis, mainly referring to the membrane shrinkage and invagination, division and encapsulation of cytoplasm, containing DNA material and organelles, forming small EVs with a diameter ranging from 100 to 5,000 nm (Phan et al., 2020). ABs can maintain homeostasis and fine-tune the life cycle of multicellular organisms, and mediate intercellular communication (Liu et al., 2018c; Zhao et al., 2021).

### 2.2 Methodology for the isolation of EVs

Various techniques can be used for the isolation and purification of EVs, such as ultracentrifugation, immunoaffinity capture, size-based isolation, and polymer precipitation (ALTINTAs and SAYLAN, 2023). Advantages, disadvantages, and methodologies of each method are discussed.

#### 2.2.1 Ultracentrifugation

Ultracentrifugation is the most popular method for EV isolation, known as the gold standard owing to its high efficiency and low cost (Livshits et al., 2015; Ludwig et al., 2018; Poupardin et al., 2024). Because of the different densities, cells, platelets and large apoptotic bodies will be separated from EVs by this method (Witwer et al., 2013). The simplicity of the procedure and no need of sample volume limitations are both significant advantages of this method. Conversely, large sample sizes may result in a lack of purity time-consuming process and lower EV yields (Clos-Sansalvador et al., 2022). Ultracentrifugation is applicable for the isolation of EVs from various body fluids. Although plasma is one of the most challenging bodily fluid samples to handle in EV associated studies, ultracentrifugation showed the highest purity of EVs compared to several commercial isolation kits (Tian et al., 2020). However, in order to achieve higher EV purity and yield, it is essential to optimize the ultracentrifugation method. An improved one-step sucrose cushion ultracentrifugation (SUC) method was developed based on the density and buffering properties of sucrose. Compared to conventional ultracentrifugation methods, this method demonstrates higher yields of EVs with better integrity and fewer protein contaminants (Gupta et al., 2018). In future applications, the combination of ultracentrifugation with various other isolation methods is highly necessary.

#### 2.2.2 Size-based techniques

Size exclusion chromatography (SEC) is a common EV isolation method of size-based techniques. SEC relies on the difference in size between EVs and other components in biological samples (Benedikter et al., 2017). Compared with ultracentrifugation and precipitation-based methods, SEC causes less morphological changes in EVs, thereby maintaining their integrity and biological activity. However, the SEC relies on extensive laboratory equipment, which is time-consuming (Kumar et al., 2024). Another popular size-based EV isolation techniques is ultrafiltration. It uses membranes with diverse pore sizes to selectively capture molecules, particles, or vesicles of specific dimensions while allowing smaller components to permeate through the filter (Xu et al., 2017). Its advantages include a simple process, no need for special equipment, and a high yield (Kim et al., 2021). However, it can lead to protein residue, which poses challenges for EV downstream analysis by proteomics. Tangential flow filtration (TFF) uses a cross-flow technique to concentrate and filter particles. Compared to ultrafiltration, TFF has better membrane permeability and is capable of preventing molecular accumulation and membrane fouling. Therefore, the separation of EVs has high yield and is suitable for large-scale research applications (Veerman et al., 2021; Visan et al., 2022).

In the past decade, size-based techniques are increasingly applied for EV isolation (Poupardin et al., 2024). In clinical samples, this technique can be effectively used for the isolation of EVs from various sources including urine, plasma, serum, and tissues. Typically, the isolated EVs could be well applied to downstream analysis (Sedej et al., 2022; Mazzucco et al., 2023; Zhang et al., 2023). Therefore, EVs isolated through SEC are commonly used for protein research. However, the complex process of size-based techniques needs simplification in the future, such as simplified dichotomic SEC, which can be applied for the bulk separation of EVs in clinical research (Guo et al., 2021).

#### 2.2.3 Immunoaffinity-based capture

Immunoaffinity-based capture relies on the distinctive identification of surface biomarkers proteins on EVs. The method can be achieved by incubating sample with magnetic beads coated with antibodies against the surface proteins (Li et al., 2017). It is typically used as a supplementary step combined with ultracentrifugation method to further purify isolated EVs (Reiner et al., 2017). The main advantage of the method is its ability to separate specific subtype of EVs, resulting in high specificity and purity (Tschuschke et al., 2020). However, immunocapture requires a large number of antibody conjugates, leading to high costs and making it unnecessary for use with large samples. Additionally, many biological materials are added during the EV separation process, making immunoaffinity-based capture difficult to apply for treatment purposes (McNamara et al., 2018). Immunoaffinity-based capture of EVs is already possible with the development of commercial kits. Most of these kits are coated with antibodies against CD9, CD63, and CD81 (Wiklander et al., 2018). This methodology is applicable for the isolation and characterization of distinct EV subtypes, enabling high-purity research. As a result of its efficiency and sensitivity, immunoaffinity-based capture has been recognized as a useful method. However, further studies should concentrate on the development of low-cost and less biological material added method.

#### 2.2.4 Polymer precipitation

Polymer precipitation is achieved by reducing the solubility of EVs using polyethylene glycol (PEG) as a medium (Konoshenko et al., 2018). Due to the simple operation and high yield, polymer precipitation has ability for processing large-scale samples, and offers the advantage of reduced analysis time (Batrakova and Kim, 2015). Currently, there are mature PEG-based commercial kits, such as ExoQuick™. Polymer precipitation is commonly used for the isolation of EVs in blood and cell culture (Poupardin et al., 2024). In addition, polymer precipitation can achieve the highest yield of EVs and genetic content, such as miRNA and mRNA, compared to ultracentrifugation and ultrafiltration methods, making it suitable for subsequent analysis (Patel et al., 2019). However, one disadvantage of the method is that other unnecessary precipitates can contaminate the separated EVs, reducing their purity and recovery rate. It is not conducive to downstream proteomics and other analytical work. To address this issue, additional purification steps should be employed to remove contaminants (Zarovni et al., 2015).

## 3 EVs in chronic kidney disease pathogenesis

**FIGURE 1:**
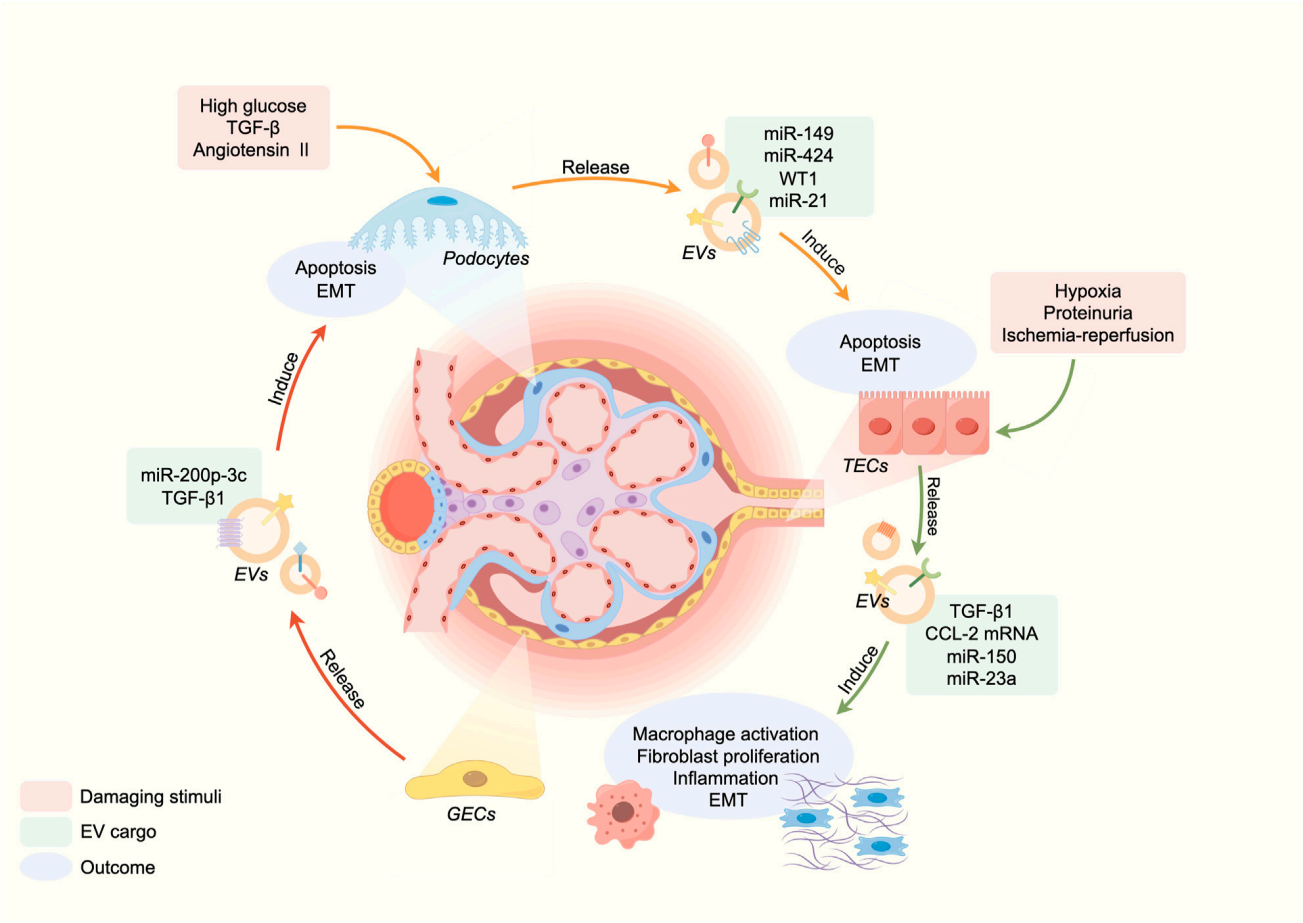
EVs in the pathogenesis of CKD.

The pathology of CKD occurs inextricably with two mechanisms: initial stimulation and persistent renal damage. Initial stimulus factors include inflammation, immune response, toxins, and underlying renal conditions. In this context, EVs participate in CKD pathogenesis through intercellular communication, facilitating content delivery and activating signaling pathways in target cells or processing pathways exclusive to cell contents (Hogan et al., 2009; Borges et al., 2013). Mechanically, EVs contribute to the pathogenesis of CKD through facilitating intercellular communication and promoting inflammation and fibrosis (Figure 1).

### 3.1 EVs in intercellular communication

Renal EVs are generated and secreted by kidney cells, and through intercellular communication, they have been linked to renal function and disorders (Krause et al., 2015). Gildea et al., 2014 demonstrated that cells in the upper segments of tubules can release EVs that can be absorbed by downstream cells, transmitting active molecules to regulate cellular behavior. Moreover, Street et al., 2011 first reported the functional transfer of AQP2 via urinary EVs in murine kidney collecting duct cells. An *in vitro* study showed that the activation of glomerular endothelial cells (GEnC) could induce the transfer of miR-200C-3p from GEnC to podocytes through EVs, affecting the communication between GEnC and podocytes (Hill et al., 2020). Similarly, EVs derived from injured podocytes may mediate crosstalk between glomeruli and tubules, inducing apoptosis in tubular interstitial cells and reducing tubular function (Jeon et al., 2020). Meanwhile, long-distance cell-to-cell communication mediated by EVs between distinct aspects of kidney could amplify kidney injury, tubulointerstitial fibrosis and inflammation (Munkonda et al., 2018; Hill et al., 2020). β-catenin is a master controller in renal tubular injury and fibrogenesis (Conduit et al., 2019; Miao et al., 2019). Chen et al. confirmed that β-catenin activated tubular epithelial cells to secrete EVs containing Osteopontin protein, which binded to CD44 receptors in fibroblasts, promoting their proliferation and activation (Chen et al., 2022b). Consequently, the crosstalk mediated by EVs is widespread in the kidney injury and the process of CKD. Regulating the intercellular communication of EVs in renal cells is a promising therapeutic strategy for CKD.

### 3.2 EVs in inflammation

Inflammation serves as a pivotal mechanism contributing to the renal damage in CKD. EVs have the potential to trigger pro-inflammatory responses, which may be attributed to the transfer of inflammatory mediators (Mesri and Altieri, 1998; Distler et al., 2006). During inflammation, both innate immune cells and damaged cells can release EVs carrying damage associated molecular patterns (DAMPs). These DAMPs can be either attached to the surface of the EVs or encapsulated into them. Meanwhile, DAMPs can activate macrophage toll-like receptors (TLRs), leading to the stimulation of NF-κB signaling and subsequent release of inflammatory cytokines and reactive oxygen species (ROS) (Cao et al., 2015). In CKD, the injury of proximal tubular epithelial cells (TECs) is closely associated with the decline in renal function. Under hypoxia condition, stimulation of proteinuria or physical damage leads to the release of various pro-inflammatory cytokines by damaged TECs, thereby eliciting an immune response (Nangaku, 2006; Liu et al., 2018b). TECs can upregulate hypoxia-inducible factor 1α (HIF-1α) and release EVs enriched with miR-23a, which induce macrophage reprogramming under hypoxia and promoting tubulointerstitial inflammation (Li et al., 2019). Albuminuria is a significant indicator of CKD and plays a vital role in the development of tubulointerstitial inflammation related to CKD (Liu et al., 2014). It is worth noting that proteinuria-stimulated renal TECs release an increased number of EVs loaded with inflammatory cytokine CCL2 mRNA, which are directly transferred to macrophages. The transfer represents a critical initial stage in albumin-induced tubulointerstitial inflammation (Lv et al., 2018b). Although many studies have been conducted on the relationship between EVs and inflammation, several unknown mechanisms still need to be explored.

### 3.3 EVs in renal fibrosis

Renal fibrosis is a crucial pathological characteristic of CKD, characterized by tubular atrophy, interstitial chronic inflammation and fibrosis, glomerulosclerosis, and vascular rarefaction. The mechanism is that renal injury leads to local fibroblast activation, continuous synthesis of extracellular matrix (ECM) proteins, resulting in ECM deposition, tissue damage, and impaired renal function (Huang et al., 2023). EVs also have a role in tubulointerstitial inflammation (Nangaku, 2006). Under hypoxia condition, damaged renal tubular epithelial cells secrete EVs containing TGF-β1 mRNA, thereby facilitating adjacent fibroblast proliferation, alpha-smooth muscle actin expression, and type I collagen production. Furthermore, extensive researches have demonstrated that the stimulation of TGF-β1 induced the release of EVs carrying miR-21 and miR-216a from TECs, thereby activating neighbor cells through the PTEN/Akt pathway to undergo epithelial-mesenchymal transition (EMT) (Zheng et al., 2018; Qu et al., 2019). In the condition of ischemia-reperfusion (IR), injured TECs were able to secrete EVs containing miR-150, which directly stimulated fibroblast and proliferation (Guan et al., 2020). The function of EVs is not only involved in initiating CKD fibrosis but also plays a key role in the progression of fibrosis is well documented. Therefore, repressing the release of EVs may thus emerge as a promising therapeutic strategy for CKD.

## 4 EVs as biomarkers of chronic kidney disease

In CKD, early diagnosis holds paramount importance for optimizing clinical treatment strategies and alleviating healthcare burdens. EVs play a crucial role in the pathogenesis and progression of CKD. Simultaneously, the potential application of EVs and their cargo as biomarkers has been accepted by researchers. Moreover, compared to other biomarkers such as urinary protein or microprotein levels, EVs do not require specific collection time. The diagnostic roles of EVs in CKD will be discussed.

The expression of miR-21 is significantly upregulated in renal tissue and closely associated with renal fibrosis (Denby and Baker, 2016). This relationship has been consistently demonstrated across various animal models of kidney disease, including unilateral ureteral obstruction (UUO) and ischemia/reperfusion (I/R) models (Zarjou et al., 2011). Moreover, the urinary EV miR-21 level was significantly increased in CKD patients, and it was positively correlated with the severity of tubulointerstitial fibrosis as well as podocyte injury (Lv et al., 2018a; Lange et al., 2019). The ability of miR-29c to reduce renal fibrosis is achieved by inhibiting EMT and decreasing extracellular matrix deposition (Chung et al., 2013), which involves multiple signaling pathways such as AMPK/mTOR (Shao et al., 2019), Wnt/β-Catenin (Huang et al., 2020) and PI3K/AKT (Feng et al., 2022). Meanwhile, several studies have demonstrated that miR-29c levels in urinary EVs were significantly decreased in patients with CKD or renal fibrosis compared to healthy people (Lv et al., 2018a; Chun-Yan et al., 2018). MiR-192 specifically expresses in renal cortical tissues (Ren et al., 2021) and is closely associated with the degree of renal fibrosis, EMT, inflammation, and oxidative stress (Bhatt et al., 2016; Ma et al., 2016). Similarly, urinary EV miR-192 was significantly increased in DN patients with proteinuria, suggesting that miR-192 could be used to distinguish between normal albuminuria and microalbuminuria and provide a reference for the early diagnosis of DN (Jia et al., 2016). However, further investigation is needed to explore the correlation between miR-192 derived from EVs and other renal function indicators. Other studies showed that urinary EV miR-181a was significant reduced in CKD patients at all stages, as well as in DN patients (Khurana et al., 2017; Zha et al., 2019; Liu et al., 2022). Furthermore, miR-181a overexpression could reduce glomerulosclerosis and tubular epithelial injury (Liu et al., 2018d).

As biomarkers, miRNA panels were important substances in EVs, which exhibit potential to develop as biomarkers in CKD. For example, Eissa et al. discovered that miR-15b, miR-34a, and miR-636 were upregulated in urinary EVs of DKD patients by PCR, and these urinary EV miRNAs were validated with 100% diagnostic sensitivity in a large sample (Eissa et al., 2016). Similarly, miR-21, miR-29c and miR-150 also can predict the fibrosis progressing in Lupus Nephritis, as a urinary EV derived multimarker panel (Solé et al., 2019). Furthermore, other RNA components in EVs, such as circRNAs can be used as urine diagnostic biomarkers for CKD (Cao et al., 2022a; Cao et al., 2022b). However, this study did not determine the relationship between urinary EV derived circRNA and kidney fibrosis, a further mechanism studiy is needed.

Proteins carried by EVs also have potential as biomarkers. In diabetic nephropathy (DN), increasing urinary EV derived AFM, CP, and IGLV7-46 were upregulated with the DN progression, regucalcin protein was significantly downregulated in both urinary EVs and kidney tissue, while increasing C-megalin protein was predictive of the progression of the albuminuric stages (Zubiri et al., 2015; De et al., 2017; Du et al., 2023). The proteome of urinary EVs has already been investigated in search of potential indicators for renal diseases, however, prospective large-scale research are necessary to confirm its accuracy.

Currently, due to the stable structure of proteins, increasing numbers of studies have applied proteomics technology to search relative proteins as biomarkers in CKD. However, compared with transcriptomics technology, proteomics technology has higher cost and more complex protocol. Thus, it is essential to establish a panel of combined biomarkers for CKD using RNAs and proteins.

## 5 Therapeutic role of EVs in chronic kidney disease

**FIGURE 2:**
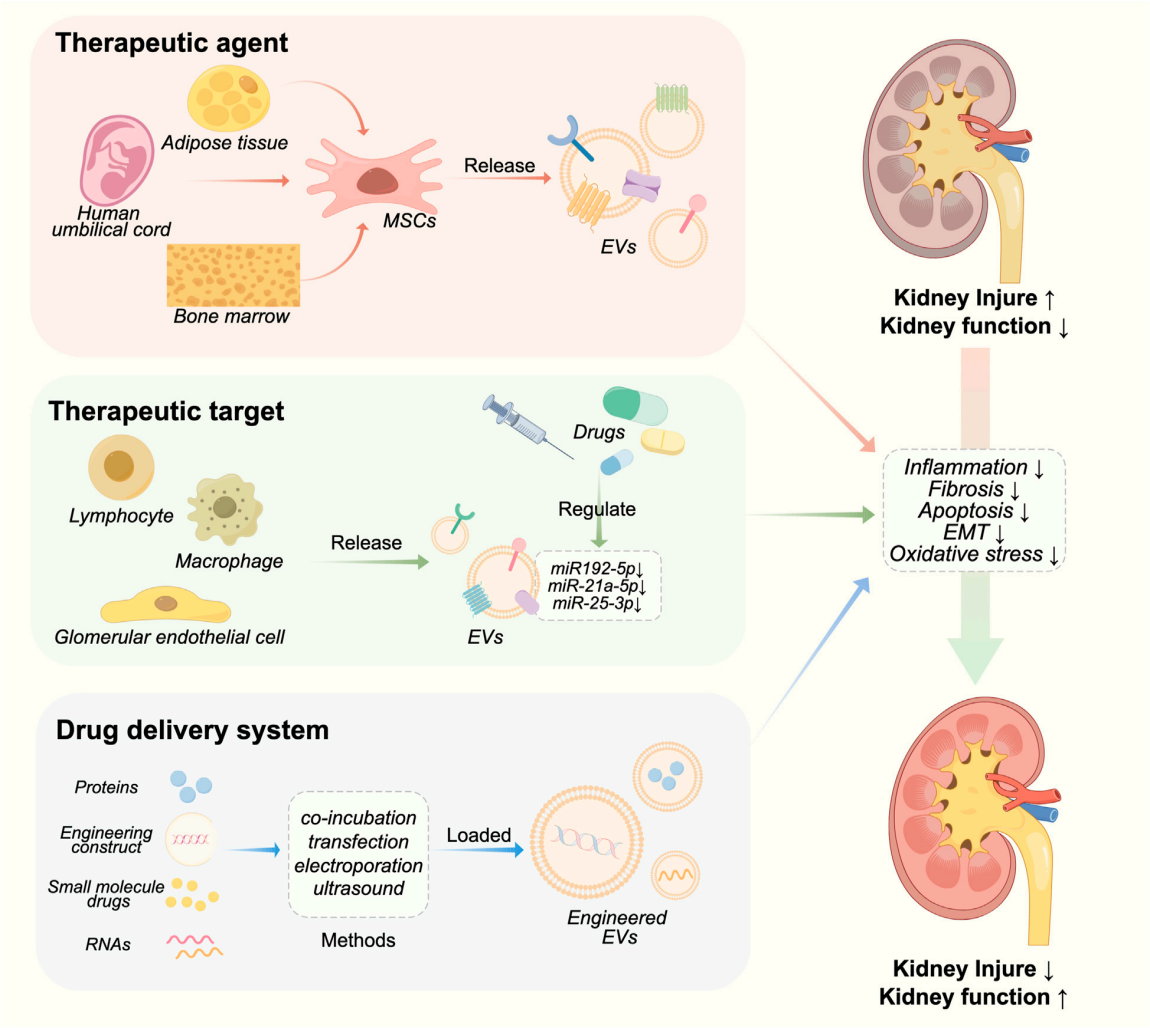
Therapeutic roles of EVs in CKD.

EV-based therapies for CKD are currently undergoing active research. EVs have shown great potential as both therapeutics and carriers for drug delivery (Table 1; Figure 2). In the field of kidney disease treatment, current research primarily focuses on the therapeutic prospects offered by EVs themselves. However, there is a lack of established systematic therapeutic regimens in this field. The therapeutic role of EVs in CKD will be discussed below, including their application as a therapeutic agent, treatment target and drug delivery carrier.

**TABLE 1:** Therapeutic roles of EVs in CKD.

Kidney disease	Models	Source of EVs	Cargos	Mechanism	Outcome	Ref.
DN	STZ-induced DN rats	Ad-MSC	miR-125a	Regulate the activation HDAC1 and ET-1	Inhibit DN progression and alleviate the symptoms	Hao et al. (2021)
RAS	porcine model of metabolic syndrome and renal artery stenosis	Ad-MSC	-	Increase reparative macrophages, upregulate the expression of IL-10	Decrease renal inflammation, attenuated renal fibrosis	Eirin et al. (2017)
DN	db/db mice	Ad-MSC	miR-486	Inhibit the Smad1/mTOR signaling pathway in podocyte	Prevent renal injury from diabetes	Jin et al. (2019)
RAS	porcine model of metabolic syndrome and renal artery stenosis	Ad-MSC	-	Decrease microvascular oxidative stress and apoptosis	Restore renal angiogenesis and microvascular architecture	Eirin et al. (2018)
CKD	UUO mice	BM-MSC	miR-374a-5p	Inhibit apoptosis by regulate MAPK6/MK5/YAP axis	Prevent the progression of renal fibrosis	Liang et al. (2022a)
CKD	UUO mice	BM-MSC	miR-21a-5p	Inhibit glycolysis in TECs by targeting PFKM	Ameliorate renal fibrosis	Xu et al. (2022)
CKD	UUO rats	BM-MSC	miR-294, miR-133	Prevent phosphorylation of SMAD2/3 and ERK1/2	Ameliorate renal fibrosis	Wang et al. (2020a)
CKD	UUO mice	BM-MSC	miR-186-5p	Downregulate the expression of Smad5	Ameliorate renal fibrosis	Yang et al. (2022)
DN	STZ-induced DN rats	BM-MSC	-	Revert oxidative stress, ER stress, inflammatory condition, and apoptosis	Inhibit DN progression	Khamis et al. (2023)
CKD	5/6 subtotal nephrectomy mice	BM-MSC	-	Prevent fibrosis, reduce interstitial lymphocyte infiltrates	Ameliorate renal injury	He et al. (2012)
CKD	UUO mice	BM-MSC	Let-7i-5p Antagomir	Regulate TSC1/mTOR signaling	Reduce renal fibrosis and improve kidney function	Jin et al. (2021)
CKD	UUO mice	Huc-MSC	miR-874-3p	Target RIPK1/PGAM5 to regulate programmed necrosis and mitochondrial division	Attenuate renal tubular epithelial cell injury and enhance repair	Yu et al. (2023)
CKD	UUO mice	Huc-MSC	CK1δ and β-TRCP	Transport CK1δ and β-TRCP system, inhibit YAP activation	Reduce collagen deposition, alleviate renal fibrosis	Ji et al. (2020)
DN	STZ-induced DN rats	Huc-MSC	miR-146a-5p	Facilitate M2 macrophage polarization by targeting TRAF6	Restore renal function in DN rats	Zhang et al. (2022b)
DN	db/db mice	Huc-MSC	miR-424-5p	inhibit high glucose-Induce apoptosis and EMT through of YAP1	Decrease cell apoptosis, inhibit EMT	Cui et al. (2022)
DN	STZ-induced DN mice	Huc-MSC	miR-451a	Inhibit cell cycle inhibitors to restart the blocked cell cycle and reverse EMT	Promote the repair of injured kidney structure and function, improve EMT	Zhong et al. (2018)
CKD	UUO mice	Primary mouse satellite cells	miR-29	Downregulate YY1 and TGF-β pathway proteins	Ameliorate skeletal muscle atrophy and attenuate kidney fibrosis	Wang et al. (2019)

### 5.1 EVs as a therapeutic agent in chronic kidney disease

Mesenchymal stem cells (MSCs) are a type of self-renewing pluripotent cells that can be derived from various tissues and organs. MSCs have the ability to prevent kidney injury by regulating the release of EVs. MSC-derived EVs can be delivered to the kidney through intraperitoneal, arteriovenous, and intraosseous routes, exerting their protective effects on the kidneys via paracrine mechanisms such as anti-fibrosis, anti-oxidative stress, anti-apoptosis, and pro-angiogenesis (Peired et al., 2016).

Bone marrow derived MSCs (BMMSCs) have been extensively investigated and are considered the primary source of therapeutic EVs (Pittenger et al., 1999; Tang et al., 2022). Zhang et al. (2022a) discovered that miR-16 and miR-21 in BMMSC EVs could induce the polarization of a specific anti-inflammatory macrophage phenotype within the kidneys of MRL/lpr mice, thus alleviating lupus nephritis. The miR-21a-5p carried by BMMSC EVs can effectively suppress the expression of phosphofructokinase muscle isoform, which is the rate-limiting enzyme in glycolysis, thereby reducing glycolytic activity in TECs. Consequently, this mechanism contributes to the amelioration of renal fibrosis in UUO mice (Xu et al., 2022).

The abundant source and low immunogenicity make human umbilical cord mesenchymal stem cell (hUCMSCs) a proper choice for allogeneic cell therapy, highlighting their suitability as a type of stem cells (Ding et al., 2018). HUCMSC EVs possess the benefits of hUCMSCs while evading their drawbacks (Huang et al., 2022). The protective effect of hUCMSC EVs on CKD mainly manifests through the regulation of inflammation and immunity. The presence of miR-22-3p in hUCMSC EVs exhibits anti-inflammatory and nephroprotective effects in diabetic mice by downregulating NLRP3 expression, while also conferring podocyte protection (Wang et al., 2023). MiR-146a-5p in hUCMSC EVs targeted the TRAF6/STAT1 pathway, thereby facilitating macrophage polarization towards the M2 phenotype and ameliorating renal injury in DN rats (Zhang et al., 2022b).

Compared to bone marrow and umbilical cord, adipose tissue provides a less invasive and more accessible source of stem cells (Mazini et al., 2020). Zhu et al., 2017 demonstrated that EVs derived from adipose-derived MSCs (ADMSCs) possessed the ability to counteract the AKI-CKD transition by suppressing the expression of sox9 in TECs. In the porcine model of metabolic syndrome and renal artery stenosis, Eirin et al. observed that ADMSC EVs exhibited multiple functions, including enhancing modulation of renal angiogenic factors expression, amelioration of kidney inflammation, improvement in medullary hypoxia and fibrosis, thereby presenting a potential therapeutic approach for addressing metabolic syndrome and renal artery stenosis (Eirin et al., 2017; Eirin et al., 2018).

### 5.2 EVs as a therapeutic target in chronic kidney disease

As previously mentioned, EVs play a pivotal role in biological processes of CKD. Consequently, they have emerged as a promising therapeutic target for the treatment of CKD. Zhuang et al., 2022 demonstrated that GABA could mitigate the pro-inflammatory effects of macrophages on podocytes by modulating miR-21a-5p/miR-25-3p in macrophage-derived EVs. Ding et al., 2021 discovered that the exosome inhibitor GW4869 effectively attenuated cyst growth in Autosomal Dominant Polycystic Kidney Disease (ADPKD) and reduced macrophage infiltration in cystic kidneys. Moreover, systemic administration of GW4869 did not induce hepatic or renal toxicity. Pharmacological inhibition of exosome biogenesis and release, exemplified by GW4869, shows promise as a potential therapeutic strategy for the treatment of ADPKD.

Numerous traditional Chinese medicines and natural products have demonstrated efficacy in ameliorating chronic kidney disease through the utilization of EVs. *Panax ginseng* saponins have the potential to alleviate steroid resistance in the mouse glomerular endothelial cells (GECs) by modulating hormone-resistant signals present in lymph-derived EVs (Chen et al., 2022a). The Jian-Pi-Yi-Shen Formula exerts a reno-protective effect in adenine-induced CKD rats by attenuating the release of miR-192-5p from macrophage-derived EVs (Liang et al., 2022b).

### 5.3 EVs as a drug delivery system in chronic kidney disease

EVs have been extensively considered as an efficient drug delivery system in recent years due to their compatibility, low toxicity, long half-life, non-immunogenicity, and effective targeting ability towards various cells (Rajput et al., 2022). There are two methods for packaging cargo into EVs, including endogenous and exogenous loading. Exogenous drug loading, which involves extracting and purifying EVs, and then encapsulating therapeutic drugs in EVs, has the advantage of simple preparation. Common methods include electroporation, co-incubation, ultrasound, chemical transfection, and repeated freeze-thaw cycles. Another method is endogenous drug loading, which involves using genetic engineering techniques or co incubation to introduce target molecules into donor cells, followed by secretion of extracellular vesicles from the donor cells, and finally recovering the drug loaded EVs through separation and purification (Gupta et al., 2021).

Sun et al. generated and isolated ADMSC-derived EVs overexpressing GNDF using lentiviral transduction, demonstrating significant attenuation of renal fibrosis in UUO mice. Moreover, these EVs exhibited the ability to enhance peritubular capillary angiogenesis following kidney injury by activating the SIRT1/eNOS pathway (Chen et al., 2020). Endogenous cargo involves genetically modifying parent cells to regulate the cargo during EVs biogenesis (Gupta et al., 2021). Wang et al., 2019 used engineered EVs vectors containing miRNA-29 and targeting peptide RVG, enabling specific renal targeting. In the fibrotic kidneys in UUO mice, these engineered EVs exhibited enhanced accumulation and effectively mitigated renal fibrosis by suppressing YY1 and TGF-β pathway proteins. This study demonstrates the feasibility of constructing therapeutic-loaded engineered EVs.

Besides, enhancing the targeting ability of EVs to kidney injury sites can significantly enhance the therapeutic effect of EVs in CKD. One strategy is the utilization of peptides or antibodies that exhibit specific binding affinity towards specific molecules. Kidney injury molecule-1 (Kim-1) is recognized as a marker for tubular injury in AKI (Vaidya et al., 2010). Tang et al., 2021 established a red blood cell-derived EVs (RBCEVs)based drug delivery platform and conjugated Kim-1-targeting LTH peptides to RBCEVs to target renal tubular injury. In addition, Wu et al. constructed a neutrophil membrane-engineered nanoparticle (NEX) that significantly promoted targeted enrichment of EVs in damaged renal tissue, thereby improving AKI (Wu et al., 2022). In addition, various hybrid approaches from tumor treatment models have provided valuable insights for targeted EV therapy in CKD. These methods include the hybridization of synthetic liposomes with EVs and the conjugation of diacyllipid-aptamer conjugates with EVs (Zou et al., 2019), providing support for precise targeted therapy of CKD.

## 6 Conclusion and perspective

EVs in the diagnosis and treatment of CKD have made a lot of advancements, thus holding immense potential for future applications. The distinctive characteristics of EVs, including their inherent stability, biocompatibility, and capacity for intercellular biomolecule transfer, render them highly appealing candidates for non-invasive diagnostics and targeted therapeutics in CKD. However, as research progresses, the methods of EV isolation and their clinical applications still require further exploration.

Despite increasing studies about EVs have been investigated, our comprehension of the EV cellular and molecular mechanisms remains limited. The technical difficulty of precisely distinguishing specific subtypes of EVs poses a significant limitation. The development of multi-omics technology provides technical basis to reveal the mechanism of EVs. Hence, we suggest the construction of a public database using multi-omics data (genome, transcriptome, proteome, and metabolome) on EVs.

EVs are currently isolated and purified using various techniques, but there are still some areas that need improvement. Firstly, there is no standardized method for EV isolation. This lack of standardization makes it challenging to compare results across studies and hinders the reproducibility of research findings. Therefore, it is important to establish standardized isolation protocols that can be widely adopted. Secondly, most isolation methods rely on the physical characteristics of EVs, which may also co-isolate other extracellular vesicles or contaminants, leading to impure EV preparations. The combined use of EV isolation methods has become a common trend in recent years (Poupardin et al., 2024). Hence, we suggest that several strategies such as the combination of isolation methods and the use of multiple markers should be used to enhance the specificity of EV isolation.

Through extensive research and experimentation, researchers have found some potential renal-specific EV-derived biomarkers that can serve as diagnostic tools for CKD in the future. The detection of these specific markers in urine samples provides valuable insights into the underlying pathophysiology and progression of CKD, enabling early intervention and personalized treatment strategies. Nonetheless, the current studies have small sample sizes, and larger clinical samples will be necessary in the future to determine the potential of specific EVs as biomarkers. Although some EV-derived biomarkers associated with CKD have been discovered, further identification and confirmation of specific biomarkers for particular diseases are necessary. This will enhance the accuracy and reliability of EVs as biomarkers for CKD.

EVs play a pivotal role in intercellular communication and the transfer of miRNAs, mRNA, proteins, and other bioactive molecules. These properties can be exploited for therapeutic purposes in CKD. Researchers have also investigated the potential of engineering EVs to delivery therapeutic cargo, including anti-inflammatory agents, growth factors, and gene-editing tools, directly to sites of renal injury. These targeted strategies have exhibited promising outcomes in preclinical investigations, demonstrating the capacity of EVs to ameliorate renal damage, facilitate tissue regeneration, and enhance overall renal function. However, EVs in the treatment of CKD still have some issues that need to be addressed. Achieving targeted delivery and ensuring specificity remains a challenge. Novel techniques and strategies such as surface modification, specific receptor recognition, and the design of targeted delivery nanoparticles, can be developed to resolve the problem. Additionally, the safety and long-term effects of EV-based therapies need to be thoroughly assessed through rigorous preclinical and clinical studies. Furthermore, the development of EV-based drug delivery systems offers great potential for individualized and targeted CKD therapy. By engineering EVs with specific surface proteins and loading them with therapeutic agents, it becomes possible to precisely target afflicted cells or tissues, minimizing off-target effects and optimizing treatment outcomes. Moreover, the integration of EVs with nanotechnology and bioengineering approaches will facilitate the development of novel platforms for controlled release and enhanced cargo delivery to the kidneys.

Currently, the use of EVs for treating CKD is still at initial stage. Research on targeted delivery of EVs is mainly focused on the heart and tumors, with limited studies related to the kidneys. Most therapeutic studies using EVs for CKD lack material science support and overlook kidney targeting, which is an important clinical objective. Therefore, we advocate that in future research directions, emphasis should be placed on the targeting of EVs to sites of renal injury.

In conclusion, with ongoing research, technological advancements, EVs have the potential to revolutionize the field of renal diagnostics and therapeutics. The ability to non-invasively detect and monitor CKD progress, coupled with targeted and personalized treatments using EV-based platforms, holds promise for improving patient outcomes and reducing the burden of CKD worldwide.
